# Tissue Regeneration and Physiological Functional Recovery in Dental and Craniofacial Fields

**DOI:** 10.3390/biom11111644

**Published:** 2021-11-06

**Authors:** Takehito Ouchi, Taneaki Nakagawa

**Affiliations:** 1 Department of Physiology, Tokyo Dental College, 2-9-18, Kanda-Misaki-cho, Chiyoda-ku, Tokyo 101-0061, Japan; 2 Department of Dentistry and Oral Surgery, Keio University School of Medicine, 35 Shinano-machi, Shinjuku-ku, Tokyo 160-8582, Japan; 3 Department of Developmental Biology, Harvard School of Dental Medicine, 188 Longwood Avenue, Boston, MA 02115, USA

Dental and oral tissues maintain homeostasis through potential reparative or regenerative processes. This native biological regulation ensures appropriate oral function and provides systemic health conditions through ideal digestion and intake of nutrition. Even if some tissues break down, general dental treatment or the administration of growth factors can help them to recover. However, a large disruption of oral tissues due to tooth loss, malignant diseases, and severe trauma can cause irreversible tissue loss in the oral cavity. To compensate for this, artificial materials, such as prostheses and dental implants, support the improvement in human quality of life. We need to consider both native biological elements and biomedical products. This Special Issue of *Biomolecules*, titled “Oral Regenerative Medicine: Current and Future”, covers the dental field and focuses on craniofacial tissue regenerative therapy. Dental pulp and periodontal tissue-derived cells are well-known as major cell sources in the dental field. Dental pulp stem cells (DPSCs) are promising cell sources for biomineral tooth complexes [1]. Pilbauerova et al. reported a detailed analysis of the effect of cultivation passaging on the relative telomere length and proliferation capacity of DPSCs [2]. The authors found that the excessive proliferation of DPSCs during in vitro culture results in telomere attrition. In vitro cultivation without passaging enabled the preservation of telomere length. A further detailed analysis of stemness, including differentiation ability and functional features, is essential to further validate this finding. DPSCs are not only useful for the regeneration of dentin pulp complexes but also for wound healing in other tissues due to their multifaceted effects [3]. In this regard, hyaluronic acid (HA) plays an essential role in wound healing [4]. Schmidt et al. demonstrated the effect of HA on DPSCs in vitro. Low-molecular-weight fragments that have been produced by the enzymatic cleavage of HA have bioactive properties that are different from those of high-molecular-weight HA [5]. The authors revealed that low-molecular-weight fragments of HA induced an acute reduction in the proliferation of DPSCs and soon recovered in subsequent passages. Low-molecular-weight fragments of HA conserved the expression of stem cell markers, such as CD29, CD44, CD73, and CD90, indicating that HA may be a useful material for scaffold tissue engineering with DPSCs. DPSCs physiologically give rise to odontoblasts, which drive dentin formation. Odontoblasts play an essential role in both dentin mineralization and sensory transduction, following the stimuli applied to the dentin surface. Kimura and Shibukawa’s group identified that plasma membrane Ca^2+^-ATPase (PMCA) in rat and human odontoblasts mediates dentin mineralization [6]. This study demonstrated that PMCA contributes to the maintenance of intracellular free Ca^2+^ homeostasis in odontoblasts. They suggested that PMCA not only plays a critical role in physiological dentin formation but also in the pathological reactionary dentin formation induced by multiple external stimuli. It is also used in the application of high-pH dental materials on the dentin surface. 

Among oral tissues, the periodontium is also permanently exposed to mechanical forces resulting from chewing, mastication, or orthodontic force. Brockhaus reported on the preservation and remodeling processes within the periodontium through periodontal ligament fibroblasts during orthodontic tooth movement. Tooth movement by mechanical stress initially decelerates the periodontal ligament fibroblast cell cycle and proliferation. After adapting to environmental changes, cells can regain periodontium homeostasis, affecting their reorganization [7]. Dieterle et al. summarized the mechanobiology of the health, pathologies, and regeneration of oral periodontal tissues [8]. Further elucidation of the cell type specific and spatiotemporal fine tuning of mechanobiological processes will be a strong tool in the field of oral regenerative medicine. Periodontal tissue development originates from dental follicle progenitor/stem cells (DFPCs). Bi and Fan’s group summarized the cell–cell interactions and signaling pathways in DFPCs [9]. For DFPC-related tissue engineering, such as alveolar bone repair, periodontal regeneration, and bio-root complex formation, an ideal scaffold is of vital importance in advanced preparation technology. As DFPCs play a unique role in maintaining a favorable microenvironment for stem cells, they are useful for applications in nervous tissue regeneration and therapies for autoimmune and inflammatory diseases as well. Stock reported an extremely rare case of classical and periodontal Ehlers–Danlos syndrome [10]. Dental diseases caused by genetic mutations can provide us with greater detail about their pathogenesis. Recent studies have revealed that genetic mutations not only show dental abnormalities, but that they also impair calvaria bone formation, resulting in neurocognitive defects due to increased intracranial pressure [11]. Central nervous system-related functional decline is also an important pathogenesis that influences oral function. Thus, it is essential to understand the systemic regulation for peripheral oral functional stability. Long bones and calvaria are pivotal tissues that can provide a better understanding of the relationship between peripheral oral and systemic regulation. Tissue regeneration and functional recovery in regenerative medicine are based on signaling pathways and molecular mechanisms. Li et al. summarized the diversity of cell signaling pathways in the calvaria suture, which is a unique place to construct the cranial bone and protect the brain [12]. 

This Special Issue suggests the importance of understanding the relationship between both the cell source and effective regeneration. Physiological functional recovery is the most essential problem and the ultimate purpose of regenerative therapy. A deeper understanding of the pathological background and genetic basis and cellular signaling in local-systemic regulation is essential in dental tissue regeneration.
